# An Ultrafast One-Step Quantitative Reverse Transcription–Polymerase Chain Reaction Assay for Detection of SARS-CoV-2

**DOI:** 10.3389/fmicb.2021.749783

**Published:** 2021-11-04

**Authors:** Jadranka Milosevic, Mengrou Lu, Wallace Greene, Hong-Zhang He, Si-Yang Zheng

**Affiliations:** ^1^Captis Diagnostics Inc., Pittsburgh, PA, United States; ^2^Biomedical Engineering Department, Carnegie Mellon University, Pittsburgh, PA, United States; ^3^Penn State Hershey Medical Center, Penn State College of Medicine, Hershey, PA, United States; ^4^Electrical & Computer Engineering, Carnegie Mellon University, Pittsburgh, PA, United States

**Keywords:** ultrafast, one-step RT-qPCR assay, SARS-CoV-2 detection, COVID-19, nasopharyngeal swab

## Abstract

We developed an ultrafast one-step RT-qPCR assay for SARS-CoV-2 detection, which can be completed in only 30 min on benchtop *Bio-Rad* CFX96. The assay significantly reduces the running time of conventional RT-qPCR: reduced RT step from 10 to 1 min, and reduced the PCR cycle of denaturation from 10 to 1 s and extension from 30 to 1 s. A cohort of 60 nasopharyngeal swab samples testing showed that the assay had a clinical sensitivity of 100% and a clinical specificity of 100%.

## Introduction

**FIGURE 1:**
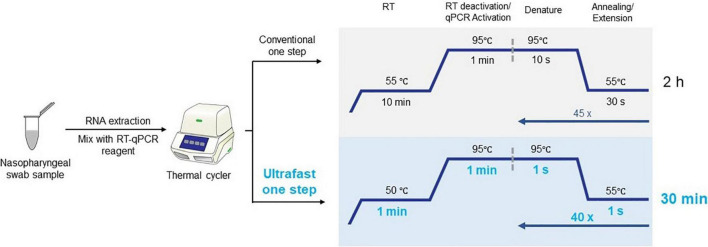
Workflow of ultrafast one-step qRT-PCR with comparison with conventional qRT-PCR for SARS-CoV-2 detection.

The current highly transmissible outbreak of severe acute respiratory syndrome coronavirus (SARS-CoV-2) is the leading cause of morbidity and mortality across the globe (Andrasfay and Goldman, 2021; Cohen, 2021; Woolf et al., 2021). Researchers have intensively invested in developing innovation for cost-effective point-of-care test kits and efficient laboratory techniques for confirmation of SARS-CoV-2 infection (Carter et al., 2020; Chen et al., 2020; Shuren and Stenzel, 2020; Venter and Richter, 2020; Wiersinga et al., 2020; El Jaddaoui et al., 2021; Mardian et al., 2021; Taleghani and Taghipour, 2021; Vandenberg et al., 2021; Yüce et al., 2021). Among those technologies, real-time quantitative reverse transcription–polymerase chain reaction (qRT-PCR) of nasopharyngeal swabs is the current gold standard in the clinical setting to confirm the clinical diagnosis of coronavirus disease 2019 (COVID-19) caused by severe acute respiratory syndrome coronavirus 2 (SARS-CoV-2) (Carter et al., 2020; Ji et al., 2020; Tang et al., 2020; Kevadiya et al., 2021). Conventional qRT-PCR for SARS-CoV-2 detection usually takes approximately 2 h on benchtop qPCR instrument, with 10 min of reverse transcription, followed with initial denaturation for 1 min, and 45 PCR cycles of 10 s denaturation and 30 s extension (Figure 1; Vogels et al., 2020). However, the ongoing COVID-19 pandemic poses substantial challenges for health-care systems and their infrastructure. Therefore, to meet the pandemic challenges, it is important to significantly shorten the turnaround time in the race for increasing the number of diagnostic tests.

## Materials and Methods

### Clinical Samples

A cohort of 60 clinical nasopharyngeal swab samples including 30 SARS-COV-2 negative and 30 SARS-CoV-2 positive sample were pre-collected and deidentified, which meets the requirement of the Institutional Review Board (IRB) Exemption 4. Those clinical nasopharyngeal swab samples were stored in virial transport media at −80°C until future use. The nasopharyngeal swab samples have been tested by a Clinical Laboratory Improvement Amendments (CLIA)-certified diagnostic laboratory with an FDA approved diagnostic kit at Penn State Health Milton S. Hershey Medical Center.

### Ultrafast One-Step Quantitative Reverse Transcription–Polymerase Chain Reaction for Severe Acute Respiratory Syndrome Coronavirus Detection

The ultrafast one-step qRT-PCR was developed using primers and probes set targeting the N1 and N2 regions in the nucleocapsid (N) gene of SARS-CoV-2 and the human RNase P gene as previously published by “United States Center for Disease Control and Prevention” (CDC) (Table 1; Centers for Disease Control and Prevention [CDC], 2020). The primers and probes for N1, nucleocapsid N2, and RNase P (RP) were purchased from Integrated DNA Technologies (IDT) and diluted as recommended. Synthetic SARS-CoV-2 RNA (ATCC, VT-3276T) was used as SARS-Cov-2 RNA standards in all condition optimization of ultrafast one-step qRT-PCR assay for detection of SARS-Cov-2. The ultrafast one-step qRT-PCR was performed as follows: the one-step qRT-PCR master mix (100 μL) was prepared according to the components in Table 2. Then, in each sample, 2 μL of SARS-CoV-2 RNA standard or extracted RNA samples were added to 8 μL of ultrafast one-step qRT-PCR master mix. Then, 10 μL of reaction solution with RNA sample and qRT-PCR master mix was loaded into 96 hard-shell PCR plates (Bio-Rad Laboratories), and the PCR plate was loaded in CFX96 Real-Time PCR detection system (Bio-Rad Laboratories). Thermal cycling conditions included 1 min reverse transcription at 50°C, 1 min at 95°C for reverse transcription deactivation and initial activation of SpeedStar HS DNA polymerase, followed by 40 cycles of 1 s denaturing at 95°C and 1 s extension at 55°C. All samples with cycle threshold (Ct) value of both N1, N2, and RP ≤ 38 were considered as positive according to CDC guidelines.

**TABLE 1:** Primers and probes for N1, N2, and RNase P (RP) (Centers for Disease Control and Prevention [CDC], 2020).

Name	Oligonucleotide sequence (5′–3′)
2019-nCoV_N1-Forward primer	GAC CCC AAA ATC AGC GAA AT
2019-nCoV_N1-Revere primer	TCT GGT TAC TGC CAG TTG AAT CTG
2019-nCoV_N1-Probe	FAM-ACC CCG CAT TAC GTT TGG TGG ACC-BHQ1
2019-nCoV_N2- Forward primer	TTA CAA ACA TTG GCC GCA AA
2019-nCoV_N2- Revere primer	GCG CGA CAT TCC GAA GAA
2019-nCoV_N2-Probe	FAM-ACA ATT TGC CCC CAG CGC TTC AG-BHQ1
RP- Forward primer	AGA TTT GGA CCT GCG AGC G
RP- Revere primer	GAG CGG CTG TCT CCA CAA GT
RP- Probe	FAM—TTC TGA CCT GAA GGC TCT GCG CG—BHQ-1

**TABLE 2:** Components of the ultrafast one-step qRT-PCR master mix.

Reverse transcription master mix			
Stock solution	Supplier	Volume/μL for 10 reactions	Final concentration
10 mM dNTPs	Thermo Fisher Scientific (R0191)	1.2	0.012 mM
5X SuperScript IV Reverse Transcriptase buffer	Thermo Fisher Scientific (18090010)	6	0.03X
100 mM DTT	Thermo Fisher Scientific (18090010)	1	0.11 mM
RNaseOUT inhibitor (40 U/μL)	Thermo Fisher Scientific (10777019)	1	4 U
SuperScript IV Reverse Transcriptase (200 U/μL)	Thermo Fisher Scientific (18090010)	1	20 U
Stabilizer Reagent	Sigma (PNS1010)	1	0.1 μL
**qPCR master mix**			
SpeedStar HS DNA polymerase (5 U/μL)	TaKaRa (RR070B)	0.8	0.4 U
Fast Buffer I (10X)	TaKaRa (RR070B)	10	0.1 X
N1 forward primer/reverse primer/probe (10 μM)	IDT	4/4/2	40 nM/40 nM/20 nM
N2 forward primer/reverse primer/probe (10 μM)	IDT		
RP forward primer/reverse primer/probe (10 μM)	IDT		
Nuclease Free H_2_O		Mix reverse transcription master mix with qPCR master mix, and then add up to 100 μL for qRT-PCR Master Mix	
qRT-PCR		Aliquot 8 μL of qRT-PCR Master Mix, and then add 2 μL of RNA template per reaction	

### FDA Approved Diagnostic Kit “Xpert^®^ Xpress SARS-CoV-2”

The Xpert Xpress SARS-CoV-2 test is an automated *in vitro* diagnostic test for qualitative detection of nucleic acid from SARS-CoV-2. The Xpert Xpress SARS-CoV-2 test was performed on GeneXpert Instrument Systems according to the protocol from the manufacturer (Loeffelholz Michael et al., 2020; Food Drug Administration [FDA], 2021).

### RNA Extraction From Nasopharyngeal Swab Samples

Total RNA was isolated from the heat inactivated nasopharyngeal swab samples using Direct-zol^TM^ RNA Microprep (R2060, Zymo Research) by following the manufacturer’s instruction. In brief, 300 μL of nasopharyngeal swab samples were lysed in 400 μL of Trizol. Then 700 μL of 100% ethanol was added, followed by column purification using Zymo-Spin^TM^ Column. Direct-zol^TM^ RNA PreWash and RNA Wash Buffer were added sequentially to wash the column. Finally, RNA was eluted in 12 μL of nuclease free water and stored in −80°C until future use.

### Statistical Analysis

Continuous and categorical variables are expressed as means (SD) and number (%), respectively, analyzed with Prism 8.0.1 (GraphPad Software, La Jolla, CA). Clinical agreements were analyzed according to Clinical and Laboratory Standards Institute (CLSI) EP12-A2 as recommended in FDA Guidelines, performed with MedCalc^®^ Statistical Software version 19.7.4 (MedCalc Software Ltd., Ostend, Belgium).

## Results and Discussion

Here, we described an ultrafast one-step qRT-PCR assay for the qualitative detection of SARS-CoV-2 that is fully compatible with conventional benchtop qPCR instruments. SARS-CoV-2 RNA was reverse transcribed for 1 min into cDNA and amplified with 40 PCR cycles of 1 s denaturing and 1 s extension step (Figure 1). This one-step qRT-qPCR assay can detect down to 25 copies of SARS-CoV-2 RNA in 10 μL reaction volume. The assay employs primers and probes developed by the United States Centers for Disease Control and Prevention (CDC) targeting N1 and N2 regions of nucleocapsid gene of SARS-CoV-2 with the internal control human RNase P gene (RP). The total ultrafast one-step qRT-PCR can be completed in 30 min on benchtop *Bio-Rad* CFX96 platform.

**FIGURE 2:**
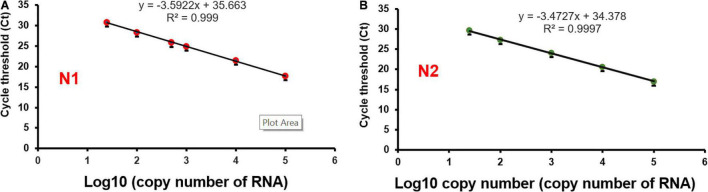
Limit of detection of ultrafast one-step qRT-PCR assay. SARS-Cov-2 synthetic RNA genome was used as a model sample. RNA templates were series diluted in the range of 25–1 × 10^5^ copies. Ultrafast one-step qRT-PCR assay detects both N1 **(A)** and N2 **(B)** regions of the nucleocapsid gene of SARS-CoV-2.

In developing the ultrafast one-step qRT-PCR assay, we reasoned that the enzymes in the qRT-PCR are key to significantly shortening the qRT-PCR and to keeping comparable sensitivity as conventional qRT-PCR for SARS-CoV-2 detection. We found that SpeedSTAR HS DNA Polymerase is optimized for PCR with extension time as fast as 10 s/kb. The amplicons of N1, N2, and RP are within the length of 100 bp. Therefore, we investigated whether the N1/N2 SARS-CoV-2 RNA could be detected with the fast PCR cycle setting of 2 s/cycle (it includes 1 s denaturing and 1 s extension step) on a conventional qPCR instrument by using SpeedSTAR HS DNA Polymerase (Giese et al., 2009). Using synthetic SARS-CoV-2 RNA from ATCC as the model, we found that 0.4 U of SpeedSTAR HS DNA Polymerase (in 10 μL of qRT-PCR reaction mixture) in the one-step qRT-qPCR assay can detect down to 25 copies of N1 and N2 of SARS-CoV-2 RNA (Figure 2). Furthermore, in RT step, we chose SuperScript IV Reverse Transcriptase because of its fast speed in cDNA synthesis (Martín-Alonso et al., 2021). We demonstrated that ultrafast one-step qRT-PCR can still detect down to 25 copies of N1 and N2 of SARS-CoV-2 RNA genome (Figure 2) by reducing the RT step from 10 min to 1 min with 20 U of SuperScript IV Reverse Transcriptase (in 10 μL of qRT-PCR reaction mixture). The limit of detection of the developed ultrafast one-step qRT-PCR is comparable to the other CDC qRT-PCR tests (Arnaout et al., 2020). During the optimization of this ultrafast one-step qRT-PCR assay, we investigated various time length of RT step (5, 2, and 1 min) and priming step (5, 2, 1, and 0 min). The result showed that there is no significant change in Ct values of N1 gene when RT step was reduced to only 1 min (Supplementary Figure 1). Furthermore, Ct values of the N1 gene decreased after removing the RT priming step (Supplementary Figure 2). Therefore, we used 1 min of RT step with any RT priming for the ultrafast one-step qRT-PCR assay. We also have investigated the various amounts of superscript IV reverse transcriptase (SSIV) in this assay. The result showed that there was no significant difference in Ct values between the SSIV concentration of 20, 50, and 80 U/10 μL reaction mixture. However, SSIV at 30 U/10 μL reaction mixture exhibited the lowest Ct for the N1 gene (Supplementary Figure 3). To lower the cost of this assay, we choose the SSIV at concentration of 20 U/10 μL in the formulation of the assay. We also have investigated the compatibility of this ultrafast one-step qRT-PCR assay with QuantStudio 7 Flex real-time PCR systems (Thermo Fisher Scientific, United States), which is also widely used in Clinical Laboratory Improvement Amendments (CLIA) certified laboratory. There were 10^4^ RNA copies of synthetic SARS-CoV-2 used and tested with the same protocol as benchtop Bio-Rad CFX96 qPCR instrument. The result showed that all N1, N2, and RNase P (RP) genes have been detected with Ct of 20–22 (Supplementary Figure 4), which is consistent with the data from the Bio-Rad CFX96 qPCR instrument. However, the qRT-PCR assay running time was 38:47 min, which is a little bit longer than the Bio-Rad CFX96 qPCR instrument. We hypothesize that the time difference is due to the slower heating and cooling speed in the QuantStudio 7 instrument. We envision that boosting heating and cooling speed of the qPCR instrument will further shorten this ultrafast one-step qRT-PCR assay to even less than 10 min. The recipe of the ultrafast one-step qPCR-PCR master mix (Table 2) and running protocol of ultrafast one-step qPCR-PCR are detailed in the Methods section.

**FIGURE 3:**
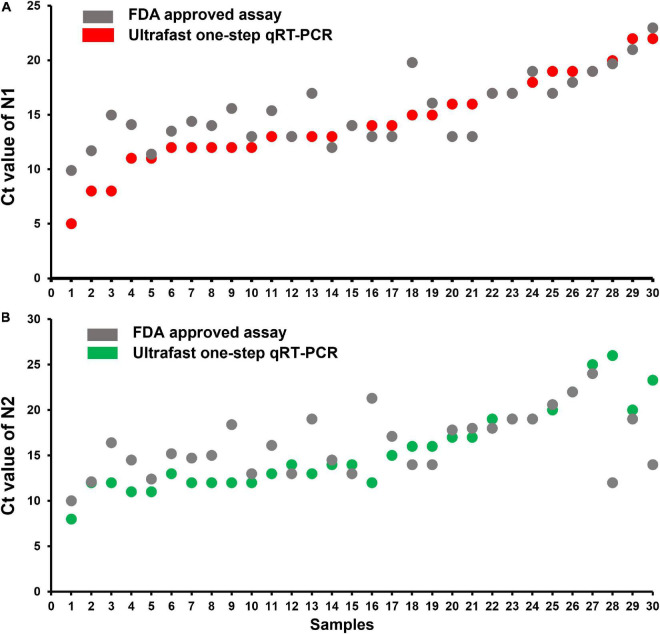
Cycle threshold (Ct values) of N1 and N2 correlation of ultrafast one-step qRT-PCR with FDA-approved assay “Xpert^®^ Xpress SARS-CoV-2” for SARS-CoV-2 positive samples. **(A)** Ct value of N1 and **(B)** Ct value of N2 from the 30 SARS-CoV-2 positive samples.

To evaluate the performance of the ultrafast one-step qRT-PCR in a clinical setting, we performed a blinded and randomized study with 30 SARS-CoV-2–positive and 30 SARS-CoV-2–negative nasopharyngeal swab samples obtained from patients. Ultrafast one-step qRT-PCR testing showed that SARS-CoV-2 positive samples exhibited N1, N2, and RNase P gene, and the cycle threshold (Ct) values of N1, N2, and RP are very close to those obtained with FDA approved diagnostics kit “Xpert^®^ Xpress SARS-CoV-2” (Figure 3 and Table 3). In SARS-CoV-2 negative samples, N1 was not detected in all negative samples, Ct values of N2 in three negative samples were above 35, which still qualifies as SARS-CoV-2 negative samples according to the CDC guidelines (Table 4). Overall, the testing results showed that the ultrafast one-step qRT-PCR had a clinical sensitivity of 100% and a clinical specificity of 100% (Table 5). Furthermore, we found that the SARS-Cov-2 viral loads in clinical samples are 3, 2 × 10^3^ – 3.0 × 10^4^ and over 6.8 × 10^4^ (Supplementary Table 1) with the standard curves for N1 (Figure 2A). The simplified format of ultrafast one-step qRT-PCR for detection of SARS-CoV-2 in nasopharyngeal swabs is suitable for use in clinical diagnostic laboratories. The limitation of this study includes that we have not explored other sample types. We will further validate ultrafast one-step qRT-PCR for SARS-CoV-2 detection in saliva samples without RNA extraction.

**TABLE 3:** Cycle threshold (Ct) value of SARS CoV-2 positive samples of ultrafast one-step qRT-PCR in comparison to an FDA approved test “Xpert^®^ Xpress SARS-CoV-2”.

	*Ultrafast one-step qRT-PCR* test		FDA approved test “Xpert^®^ Xpress SARS-CoV-2”	
Positive samples	Ct of N1	Ct of N2	Ct of N1	Ct of N2
1	12.3	12.8	13.5	15.2
2	12.7	13.4	15.4	16.1
3	14.6	15.5	19.8	21.3
4	8	11.7	11.7	12.1
5	10.6	11.1	14.1	14.5
6	5.3	7.9	9.9	10
7	12.2	12.2	14.4	14.7
8	15.4	16.4	14	14.5
9	13.5	26	12	12
10	15.2	15	16.1	17.1
11	20.1	20.3	19.7	20.6
12	17.1	17	17	17.8
13	17.1	16.7	19	19
14	14.7	14.8	13	14
15	7.7	12.5	15	16.4
16	10.9	10.7	11.4	12.4
17	15.9	16.4	13	14
18	22.3	22.1	21	22
19	22.4	24.7	23	24
20	18.9	18.8	17	18
21	19.1	19.4	18	19
22	12.2	12	14	15
23	14.2	13.6	13	13
24	13.5	13.6	13	13
25	19	18.9	19	19
26	13	12.2	15.6	18.4
27	15.9	15.8	13	14
28	16.8	17	17	18
29	12.2	12.1	13	13
30	13.1	13	17	19

**TABLE 4:** Cycle threshold (Ct) value of SARS CoV-2 negative samples of the ultrafast one-step qRT-PCR.

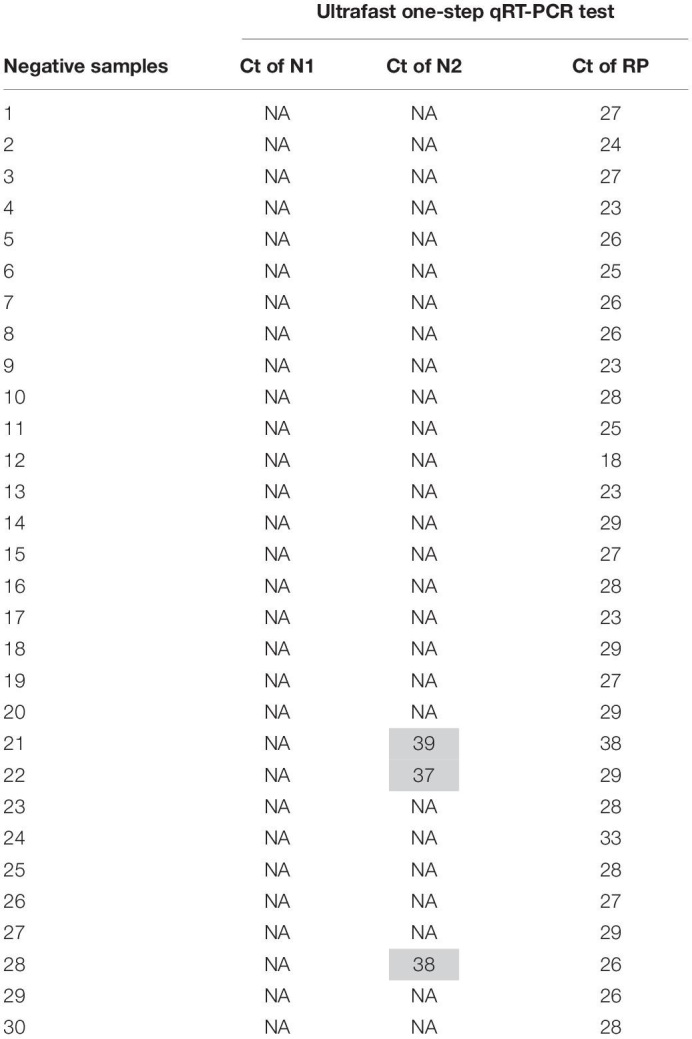

**TABLE 5:** Positive and negative predictive values of ultrafast one-step qRT-PCR for SARS-CoV-2 detection in nasopharyngeal samples.

		Comparator Assay (FDA approved assay “Xpert^®^ Xpress SARS-CoV-2”)	
		Positive	Negative
Ultrafast One Step qRT-PCR	Positive	30	0
	Negative	0	30
	Total	30	30
Percent Positive Agreement (PPA)		30/30 = 100% (95% CI: 88.7–100.0%)	
Percent Negative Agreement (PNA)		30/30 = 100% (95% CI: 88.7–100.0%)	
Percent Overall Agreement (POA)		60/60 = 100% (95% CI: 94.0–100.0%)	

## Conclusion

In summary, we have developed an ultrafast one-step qRT-PCR assay for COVID-19 diagnosis, which had a significantly reduced running time of RT and PCR step compared to conventional qRT-PCR. We further demonstrated that the ultrafast one-step qRT-PCR exhibits a limit of detection for SARS-CoV-2 that is comparable to other CDC qRT-PCR assays. Importantly, this ultrafast one-step qRT-PCR has been validated to have a clinical sensitivity of 100% and a clinical specificity of 100% with a cohort of 60 SARS-CoV-2 nasopharyngeal swab samples. We hypothesize that the success of this assay is due to the characteristics of the SpeedSTAR HS DNA Polymerase, which synthesizes new DNA strands with a speed of 10 s/kb. We envision that the high speed and high fidelity of DNA polymerase will result in fast and accurate pathogen diagnosis assay.

Furthermore, this ultrafast protocol is faster than most of the current SARS-CoV-2 detection. However, due to the limit of heating and cooling speed of the current benchtop qPCR instrument, the ultrafast one-step qRT-PCR assay protocol still takes around 30 min. We envision that boosting the heating and cooling speed of qPCR instrument will further shorten this ultrafast one-step qRT-PCR assay to less than 10 min, which will be much faster than the *Accula*^TM^ *System for SARS-CoV-2 test*. Additionally, the throughput of the Accula^TM^ System for SARS-CoV-2 test is limited to 2 samples per run, which is significantly less than 96 and/or 384 samples per run in this ultrafast one-step qRT-PCR assay. Compared with RT-LAMP, this ultrafast one-step qRT-PCR assay achieved 100% clinical sensitivity and specificity, which is much better than that of RT-LAMP with reported specificity (98%) and sensitivity (87%) (Baba et al., 2021). As such, we believe this work would be of interest to the general healthcare audience, especially those in the field of virus detection.

## Data Availability Statement

The original contributions presented in the study are included in the article/Supplementary Material, further inquiries can be directed to the corresponding author/s.

## Author Contributions

JM, H-ZH, and S-YZ designed the research. JM and WG performed research. JM and ML analyzed the data. JM, H-ZH, and S-YZ wrote the manuscript with input from all authors.

## Conflict of Interest

There is patent pending on the ultrafast one-step qRT-PCR assay for pathogen detection (US63/178797) method used in this work. S-YZ declares a competing interest in the form of consulting for and equity ownership in Captis Diagnostics. JM and H-ZH are employed by Captis Diagnostics Inc. The remaining authors declare that the research was conducted in the absence of any commercial or financial relationships that could be construed as a potential conflict of interest.

## Publisher’s Note

All claims expressed in this article are solely those of the authors and do not necessarily represent those of their affiliated organizations, or those of the publisher, the editors and the reviewers. Any product that may be evaluated in this article, or claim that may be made by its manufacturer, is not guaranteed or endorsed by the publisher.
