# Supplementary material for: An Ultrafast One-Step Quantitative Reverse Transcription–Polymerase Chain Reaction Assay for Detection of SARS-CoV-2
Source: Front Microbiol. 2021 Nov 4;12:749783. doi: 10.3389/fmicb.2021.749783 (PMC8600229; doi:10.3389/fmicb.2021.749783)
Supplement: Supplementary file 1 [file Data_Sheet_1.pdf]

Supporting information

An Ultrafast One-Step qRT-PCR Assay for Detection of SARS-CoV-2

Jadranka Milosevic[1,2], Mengrou Lu[2], Wallace Greene[3], Hong-Zhang He[1,2*], Si-Yang Zheng[2,4*]

[1]Captis Diagnostics Inc, Pittsburgh, PA, 15213.

[2] Biomedical Engineering Department, Carnegie Mellon University, Pittsburgh, PA, 15213.

[3]Penn State Hershey Medical Center and Penn State College of Medicine, Hershey, PA, 17033.

[4]. Electrical & Computer Engineering, Carnegie Mellon University, Pittsburgh, PA, United States

[*] To whom correspondence may be addressed. Email: simonhe@captisdx.com and siyangz@andrew.cmu.edu

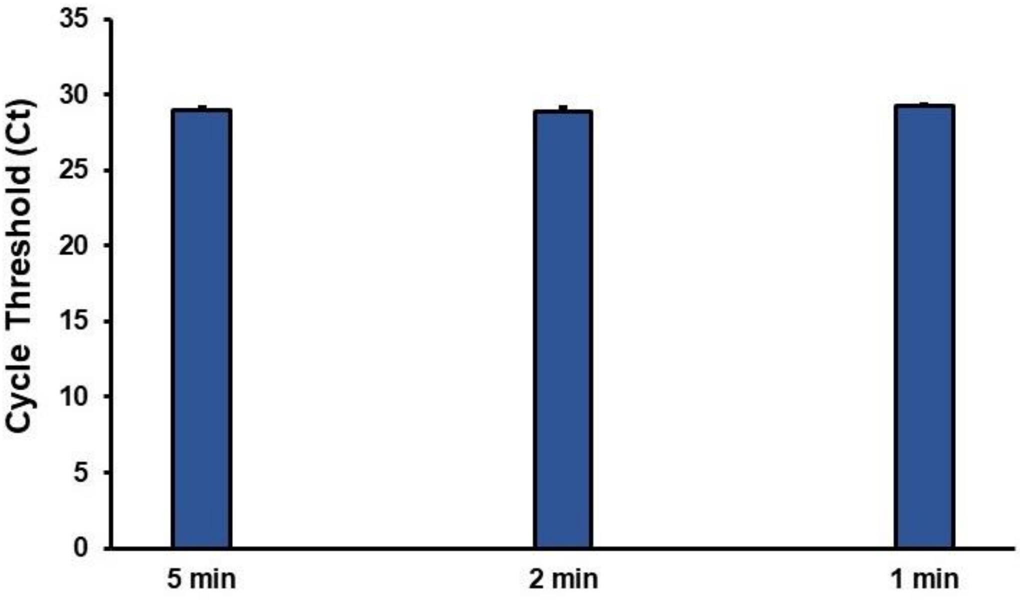

Figure S1. Optimization of the RT step for the ultrafast one-step qRT-PCR assay of on SARS-CoV-2 detection: various time length of the RT step (5, 2, and 1 minutes) with 5 minutes of RT priming (65 °C). Synthetic SARS-CoV-2 RNA (10^2 RNA copies) was used as the template in this optimization. The results are presented as the Ct value of N1 gene.

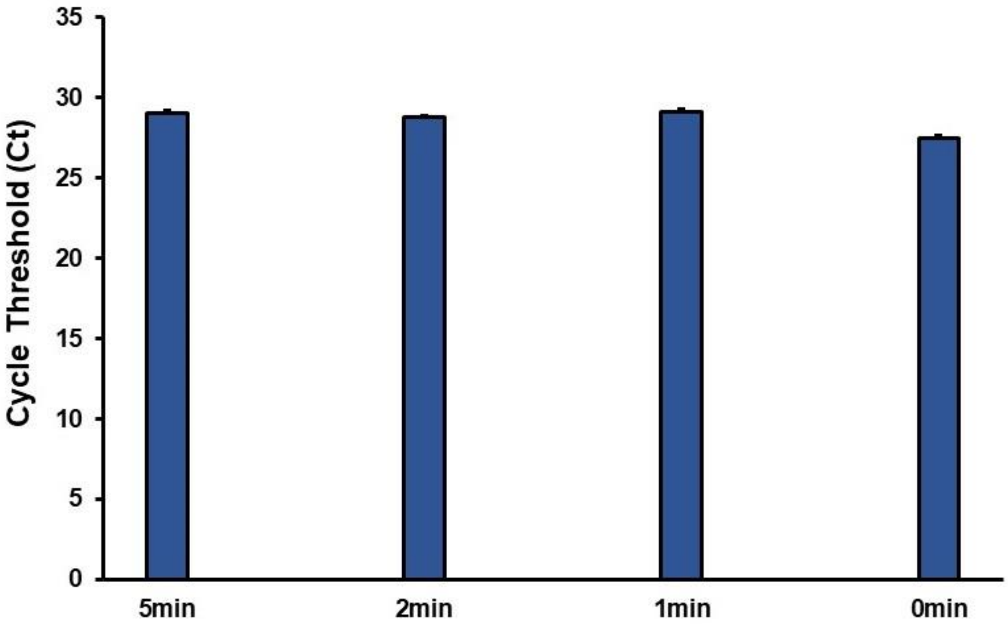

Figure 2. Optimization of the RT priming step for the ultrafast one-step qRT-PCR assay of on SARS-CoV-2 detection: various time length of RT priming step (5, 2, 1, and 0 minutes at 65 °C) with 1 minutes of RT step. Synthetic SARS-CoV-2 RNA (10^2 RNA copies) was used as the template in this optimization. The results are presented as the Ct value of N1 gene.

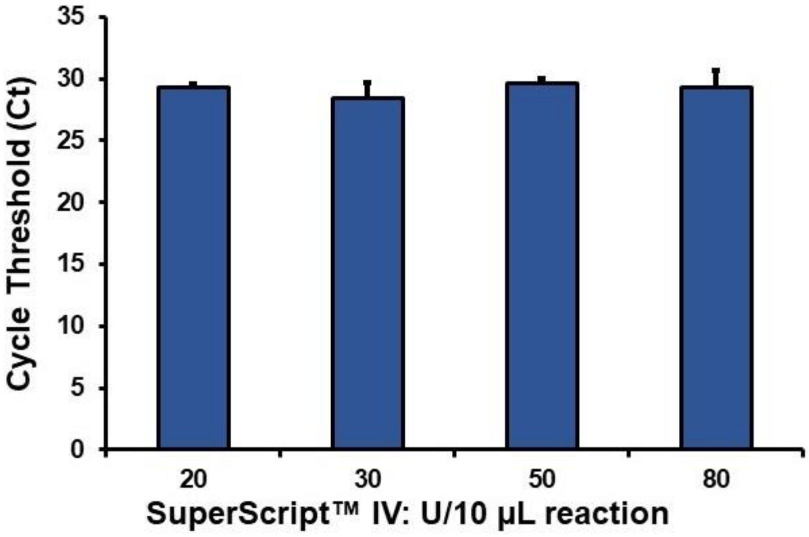

Figure 3. Optimization of the SuperScript™ IV Reverse Transcriptase amount for the ultrafast one-step qRT-PCR assay of on SARS-CoV-2 detection: various amounts of superscript IV reverse transcriptase (SSIV) in this assay (20, 30, 50, and 80 U/10 µl reaction mixture). Synthetic SARS-CoV-2 RNA (10^2 RNA copies) was used as the template in this optimization. The results are presented as the Ct value of N1 gene.

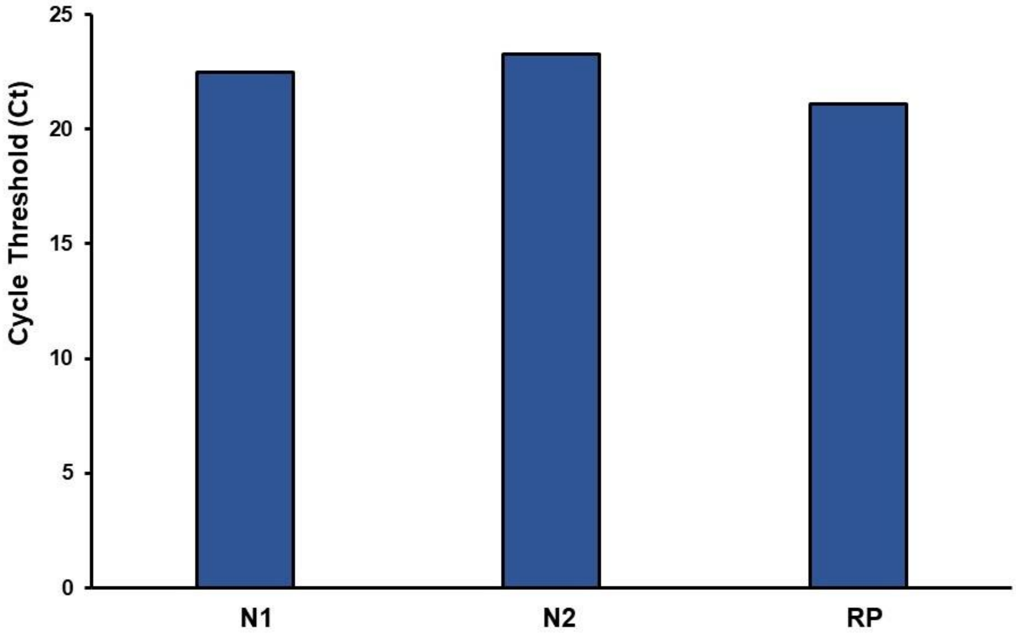

Figure S4. Ultrafast one-step qRT-PCR assay for SARS-Cov-2 detection on QuantStudio 7 Flex real-time PCR systems (ThermoFisher Scientific, US). 10^4 RNA copies of synthetic SARS-CoV-2 was used as the template and tested with the same protocol as benchtop Bio-Rad CFX96 qPCR instrument.

Table S1. SARS CoV-2 RNA Copy numbers in positive samples detected by ultrafast one-step qRT-PCR.

Samples ID	Viral Copies
1	$>6.8\times10^4$
2	$>6.8\times10^4$
3	$>6.8\times10^4$
4	$>6.8\times10^4$
5	$>6.8\times10^4$
6	$>6.8\times10^4$
7	$>6.8\times10^4$
8	$>6.8\times10^4$
9	$>6.8\times10^4$
10	$>6.8\times10^4$
11	$>6.8\times10^4$
12	$>6.8\times10^4$
13	$>6.8\times10^4$
14	$>6.8\times10^4$
15	$>6.8\times10^4$
16	$>6.8\times10^4$
17	$>6.8\times10^4$
18	$3,4\times10^3$
19	3.2×10^3
20	3.0×10^4
21	2.68×10^4
22	$>6.8\times10^4$
23	$>6.8\times10^4$
24	$>6.8\times10^4$
25	$>6.8\times10^4$
26	$>6.8\times10^4$
27	$>6.8\times10^4$
28	$>6.8\times10^4$
29	$>6.8\times10^4$
30	$>6.8\times10^4$
